# Reform of Mental Health care in Egypt, Telepsychiatry perspective

**DOI:** 10.1192/j.eurpsy.2023.1823

**Published:** 2023-07-19

**Authors:** S. I. Noby

**Affiliations:** Research department, General Secretariat of Mental Health and Addiction Treatment-MOH, Egypt, Cairo, Egypt

## Abstract

**Introduction:**

The World Health Organization (WHO) declared the outbreak of a new coronavirus disease to be a Public Health Emergency of International Concern. The situation now in Egypt, through the efforts done by the General Secretariat of Mental Health and Addiction Treatment (GSMHAT) which is governmental body following the Ministry of Health and Population: Establishment of 2 Hot lines for psychological counseling and Psychosocial support to working 24 hours/ 7 days a week. In the last year, the Mental Health platform is a main objective to be dedicated for Egyptian population, the national mental health platform is another milestone in the innovative digital remote MH services that is planned to be provided.

**Objectives:**

Evaluating the effectiveness and feasibility of different mental health services provided by hospitals affiliated to General Secretary of Mental Health and Addiction Treatment, Ministry of Health during the COVID-19 pandemic outbreak including evaluation of remote delivery of mental health service and digital health solutions.

**Methods:**

Observational Cross-sectional study. All patients seeking the hotline service & National Mental Health Platform, Evaluating the effectiveness and feasibility of services delivered over 12 months.

**Results:**

The platform’s statistical results since its launch showed that the number of visitors to the site is 44,105 and the number of registered users is 17,153. The number of self-requests conducted by users reached 16,318 and the number of virtual treatment sessions reached 2,797. The results also showed that the number of female users is 31%, compared to 69% of male users. The statistics showed that most of the age groups used for the site are between 15-20 years old, and that the users of the site are from all governorates, but most of the users are from the capital, Cairo. The statistical results also showed that general psychiatry with all its diseases is the most visited by the user of the site, followed by child and adolescent psychiatry subspecialty, then addiction.The GSMHAT, through the hotline, has provided services to the public represented by a number of calls ranging from April 2020 to March 2021 (8618) calls, and the GSMHAT hope that the hotline service to continue providing psychological counseling and support to those who wish, while continuing providing the service in hotline with the corresponding technical development and community needs.

**Conclusions:**

it is crucial to reallocate resources in order to serve for psychological and social needs and liaison psychiatry and digital as well as defining the barriers for providing a convenient service delivered by healthcare providers to people who are in need.

**Disclosure of Interest:**

None Declared

